# Spontaneous Hepatic Hemangioma Rupture in a Normotensive Twin Pregnancy: A Case Report

**DOI:** 10.7759/cureus.99128

**Published:** 2025-12-13

**Authors:** Caio Albuquerque, Beatriz Takayasu, André Ibrahim David, Juliana S Gomes, Andre Luis Dal Corso, Nicolau D'Amico Filho, Pedro C Ravizzini, Aylsa Cleyde A Queiroga, José Renato Neves

**Affiliations:** 1 Escola Paulista de Medicina (Paulista School of Medicine), Universidade Federal de São Paulo, São Paulo, BRA; 2 Surgical Gastroenterology, Hospital Samaritano Higienópolis, São Paulo, BRA; 3 Obstetrics, Hospital Samaritano Higienópolis, São Paulo, BRA; 4 Interventional Radiology, Faculty of Medical Sciences of Santa Casa de Misericordia de São Paulo, São Paulo, BRA; 5 Urology, Faculty of Medical Sciences of Santa Casa de Misericordia de São Paulo, São Paulo, BRA; 6 Cardiology, Hospital Samaritano Higienópolis, São Paulo, BRA

**Keywords:** case report, emergency cesarean section, hepatic hemangioma, spontaneous hepatic rupture, twin pregnancy

## Abstract

We report the case of a 36-year-old woman, at 28 weeks of twin pregnancy, with no history of gestational hypertensive disorders, admitted to the emergency department with intermittent chest and epigastric pain while hemodynamically stable. Upper abdominal ultrasound revealed free fluid in the abdominal cavity and hemangiomas in the right hepatic lobe. The patient subsequently developed a drop in hematimetric levels and underwent an emergency cesarean section, which confirmed intrauterine fetal demise of one twin and delivery of the surviving neonate. During subsequent exploratory laparotomy, rupture of the right hepatic lobe with a large capsular hematoma was detected, followed by hepatic packing, abdominal drainage, and reoperation after 48 hours, which achieved hemostatic control. Both the patient and the newborn were discharged after 40 days of hospitalization and were in good general condition after 4 months of follow-up.

Due to its wide variability in clinical presentation, spontaneous hepatic rupture during pregnancy is often underdiagnosed, compromising clinical management and negatively impacting maternal-fetal survival. Early diagnosis and individualized management strategies, combined with an interdisciplinary approach, are crucial for optimal maternal and fetal outcomes.

## Introduction

Hepatic hemangiomas are the most common benign tumors of the liver and are usually asymptomatic, with rare complications depending on the size and location of the lesions [1]. Although uncommon, spontaneous hepatic rupture (SHR) of hemangiomas may occur, particularly during pregnancy, and is most frequently associated with hypertensive disorders such as preeclampsia or HELLP (hemolysis, elevated liver enzymes, low platelets) syndrome [2,3].

The increased risk of SHR during pregnancy may be associated with accelerated lesion growth due to elevated estrogen levels, in addition to increased intra-abdominal pressure and direct contact with the gravid uterus [4,5]. SHR in pregnancies without hypertensive complications is rare, with only a few cases reported in the literature [6].

Management of SHR during pregnancy involves the induction of labor or emergency cesarean section, followed by immediate exploratory laparotomy. Surgical approaches vary according to intraoperative conditions and may include anatomic or non-anatomic resection, transcatheter arterial embolization (TAE), or liver transplantation [7,8]. In obstetric DIC, excessive and uncontrolled coagulation can lead to the consumption of coagulation factors, and if fibrinolysis is activated excessively, it precipitates bleeding, generating the critical threshold between thrombosis and hemorrhage [9].

This study aims to report a rare case of SHR secondary to hemangioma rupture during a normotensive twin pregnancy, successfully treated with hepatic packing and abdominal drainage. This case report follows the SCARE (Surgical CAse REport) criteria [10].

## Case presentation

A 36-year-old woman with polycystic ovary syndrome, at 28 weeks of twin pregnancy achieved by in vitro fertilization, without hypertensive disorders up to that point, presented to the emergency department of the Hospital Samaritano (São Paulo- Brazil) with intermittent chest and epigastric pain radiating to the back, right hypochondrium, and right shoulder, associated with nausea, vomiting, and dyspnea. She was hemodynamically stable, with normal vital signs: blood pressure of 128/77 mmHg, heart rate of 77 bpm, and oxygen saturation of 100%. Initial laboratory tests showed mild thrombocytopenia (160,000/mm³) and normal hemoglobin (13.7 g/dL). Twin obstetric ultrasound demonstrated both fetuses with normal heartbeats and no evidence of fetal distress or acute obstetric complications.

Acute pancreatitis was considered in the differential diagnosis; however, normal amylase/lipase levels, stable vital signs, and the absence of abnormalities on the initial abdominal ultrasound made this diagnosis unlikely. The chest pain protocol was initiated, and the results of the electrocardiogram and troponin excluded acute coronary syndrome. Physical examination revealed no signs of hypertensive disease of pregnancy, such as edema, hypertension, or hyperreflexia. Premature labor was considered, as pain episodes seemed to coincide with uterine contractions.

Within ten hours of admission, the patient developed a significant hemoglobin drop (from 13.7 to 9.3 g/dL), tachycardia (130 bpm), hypotension (80/50 mmHg), and worsening abdominal pain. Laboratory tests showed elevated alkaline phosphatase (ALP; 132 U/L), aspartate aminotransferase (AST; 148 U/L), and alanine transaminase (ALT; 231 U/L). Repeat fetal Doppler ultrasound revealed normal heart activity, with only a mild (5%) alteration in the pulsatility index of the middle cerebral artery in one fetus.

**Figure 1:**
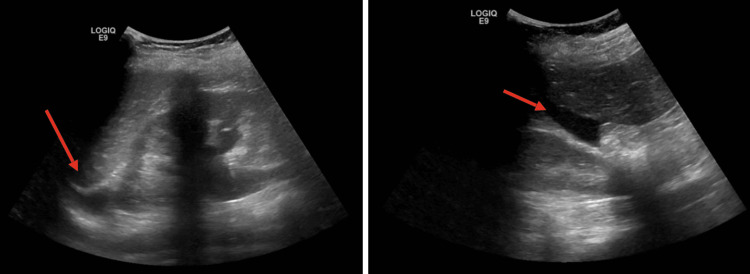
Doppler ultrasound of the upper abdomen shows hyperechogenic nodular images with imprecise limits and the presence of free fluid in the hepatorenal window

Bedside abdominal ultrasound revealed hepatic nodules (Figure 1) compatible with hemangiomas (hyperechoic lesions with ill-defined margins, no Doppler flow, largest in segment VI, 6.5 cm, heterogeneous with hypoechoic center) and a small amount of free fluid in the hepatorenal fossa (Morison’s pouch).

An emergency cesarean section was performed to preserve fetal viability. The fetus with abnormal Doppler findings showed no vital signs, confirming intrauterine fetal demise, while the surviving twin was delivered in good condition. No active pelvic bleeding was observed. Following uterine repair, exploratory laparotomy was performed, revealing right hepatic lobe rupture with a large bilobar capsular hematoma. Hemostasis was attempted using electrocautery, X-shaped sutures, hepatic packing with 10 gauze pads, and peritoneal drainage. A planned re-operation was scheduled for 48 hours later.

In the immediate postoperative period, the patient required transfusion of blood products, coagulation factors, vasoactive drugs, and aggressive fluid resuscitation for shock. Mechanical ventilation was maintained due to respiratory failure caused by fluid redistribution.

**Figure 2:**
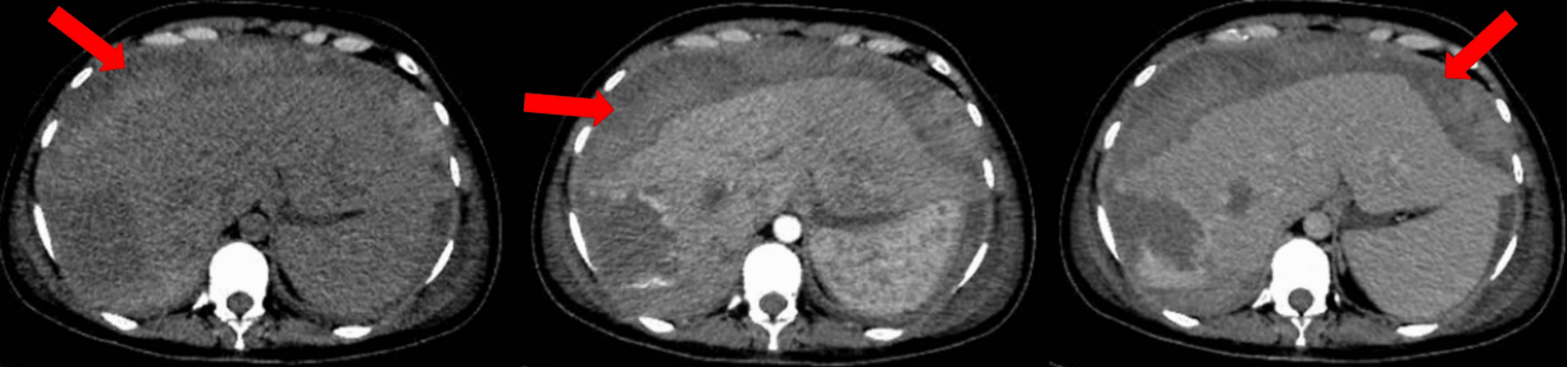
Postoperative computed tomography shows a large hepatic subcapsular hematoma

At the second-look surgery, the abdominal packs were removed without evidence of active bleeding. Absorbable hemostatic agents (SURGICEL^®^, Ethicon, Raritan, New Jersey, US) were applied, and a Blake drain (size 19; Ethicon) was placed. Postoperative CT scans revealed a large subcapsular hepatic hematoma (estimated volume 1133 mL, maximum thickness 4.8 cm) with compression of adjacent parenchyma and residual heterogeneous lesions compatible with hemangiomas (Figure 2).

**Figure 3:**
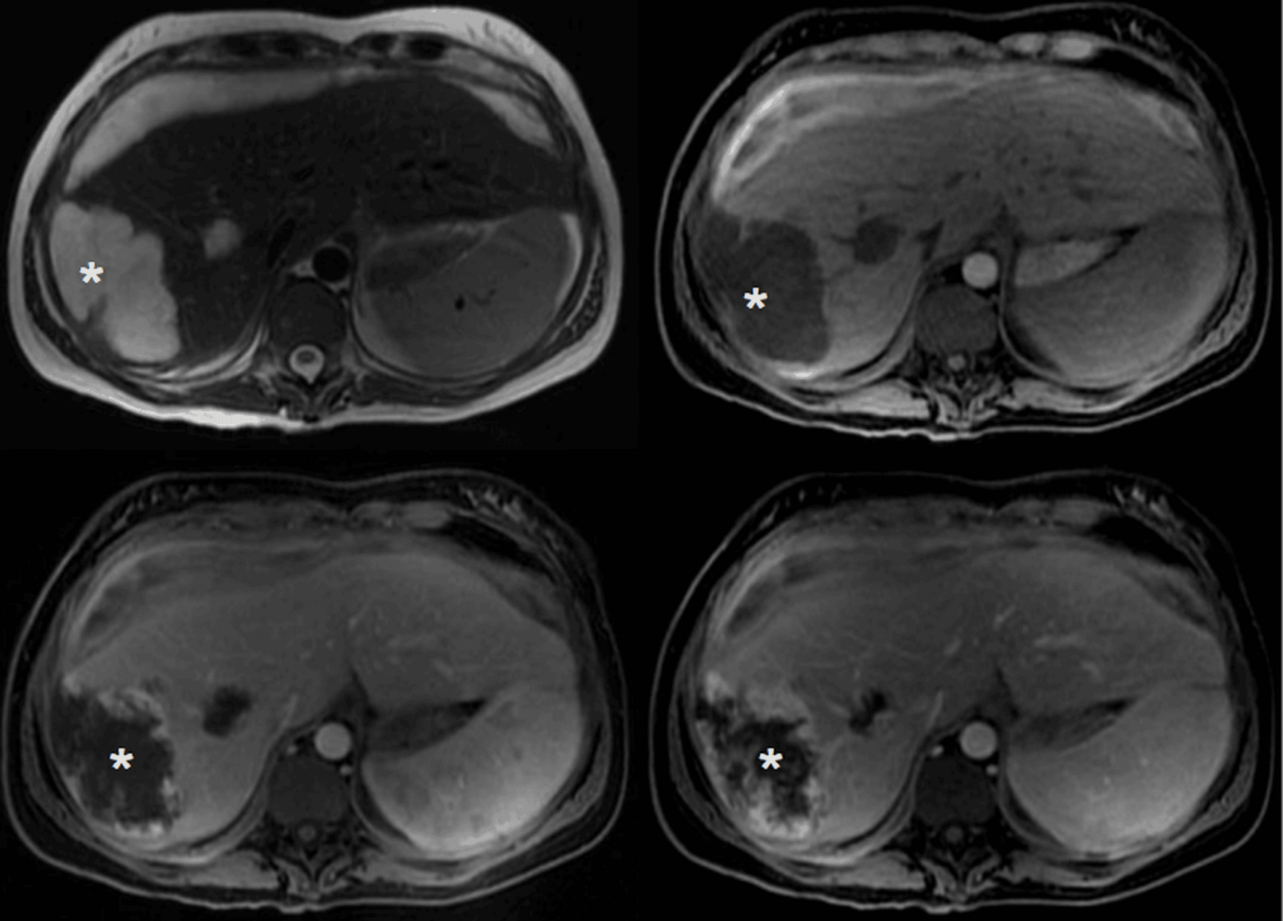
Magnetic resonance imaging shows hepatic subcapsular hematoma and hemangiomas in the right hepatic lobe

The patient remained in the intensive care unit (ICU) in critical condition, with suspected infectious complications (surgical site, ventilator-associated, or puerperal). She received ceftriaxone 2 g IV every 24 hours and metronidazole 500 mg IV every 8 hours, later escalated to meropenem 1 g IV every 8 hours and vancomycin (initial 20 mg/kg loading dose with subsequent adjustments based on renal function) after persistent fever. Hemodialysis was initiated due to persistent volume overload, and the patient underwent two sessions until achieving euvolemia. On postoperative day 15, she developed pulmonary thromboembolism (PTE) and was started on warfarin therapy, with caution due to the hemorrhagic risk from persistent hemangiomas. Magnetic resonance imaging performed in the last weeks of hospitalization demonstrated hepatic subcapsular hematoma and hemangiomas in the right hepatic lobe (Figure 3).

The therapeutic approach was effective, with control of hepatic bleeding. The patient was discharged after 42 days of hospitalization. The surviving newborn developed mild intracranial hemorrhage, with no need for drainage, and required 40 days in the neonatal ICU plus 4 days in the pediatric ward. After four months of follow-up, both mother and child were in good clinical condition, without recurrence or complications.

## Discussion

As illustrated by this case, SHR during pregnancy is an extremely rare but life-threatening condition, posing risks to both mother and fetus. During pregnancy, it is usually associated with hypertensive disorders such as preeclampsia, eclampsia, or HELLP syndrome. SHR in normotensive pregnancies is even rarer, though reported [6]. Estrogen has been shown to modulate endothelial cell behavior, promoting angiogenesis through enhanced adhesion, proliferation, migration, and capillary-like structure formation. During pregnancy, these effects are intensified, leading to the transient appearance or enlargement of hepatic hemangiomas, which may regress after hormonal levels decrease [11]. Additional risk factors include elevated intra-abdominal pressure and mechanical contact with the gravid uterus [4,5].

Symptom presentation can mimic other conditions, especially in otherwise uncomplicated pregnancies [7]. The wide variability in clinical manifestations often leads to diagnostic delays and therapeutic challenges. In a review of 391 cases, Augustin et al. reported that upper abdominal pain (83.3%), hemodynamic instability (62.4%), nausea/vomiting (24.5%), and shoulder pain (13.2%) were the most common symptoms, with nearly half of the patients presenting at least one [12].

In the present case, the absence of clear hypertensive signs delayed suspicion, with only moderate thrombocytopenia and elevated liver enzymes resembling an atypical HELLP-like syndrome. Differential diagnoses included preterm labor, acute cholecystitis, perforated ulcer, acute appendicitis, acute pancreatitis, myocardial infarction, and pulmonary embolism [3,12]. The radiation of pain to the right shoulder and back suggested acute coronary syndrome, likely due to subcapsular hematoma progression with phrenic nerve compression, while epigastric pain initially mimicked pancreatitis.

Imaging plays a key role in the diagnosis. Ultrasound is the first-line tool, but findings are variable and nonspecific; thus, CT or MRI is recommended for confirmation [3,12]. Once SHR is diagnosed, immediate intervention is required. Management generally involves cesarean delivery followed by exploratory laparotomy. Surgical strategies include hepatorrhaphy with hemostatic devices, perihepatic packing, or hepatectomy [8]. Conservative management may be considered in stable patients with an intact hepatic capsule, and laparoscopic drainage may have a role in selected cases [13].

In this case, perihepatic packing combined with intraoperative hemostatic measures achieved bleeding control. Postoperatively, pulmonary thromboembolism complicated management due to the high risk of re-bleeding under anticoagulation. The pathophysiology involves pregnancy-induced hypercoagulability and fibrinolytic activation, which, when bleeding occurs, contribute to consumptive coagulopathy and worsening hemorrhage [9].

## Conclusions

This report emphasizes that hepatic rupture, though rare and diagnostically challenging, must be considered in the differential diagnosis of pregnant women presenting with acute abdominal pain. Multidisciplinary collaboration and advanced imaging are essential for timely diagnosis and effective management. Importantly, SHR may occur independently of hypertensive disorders.
